# Blindness and visual impairment: quality of life and accessibility in the city of Turin

**DOI:** 10.3389/fmed.2024.1361631

**Published:** 2024-03-21

**Authors:** Alessia Nuzzi, Alice Becco, Andrea Boschiroli, Andrea Coletto, Raffaele Nuzzi

**Affiliations:** ^1^Eye Clinic, San Luigi Gonzaga University Hospital, University of Turin, Turin, Italy; ^2^Eye Clinic, Città della Salute e della Scienza University Hospital, University of Turin, Turin, Italy; ^3^Eye Clinic, Department of Neuroscience, San Luigi Gonzaga University Hospital, University of Turin, Turin, Italy

**Keywords:** visual impairment, quality of life, user-friendliness, architectural barriers, standard of living, visual aids

## Abstract

**Purpose:**

Despite the increase in socio-health conditions and, in general, the focus on health worldwide, many diseases still adversely affect the quality of life (QoL), including those causing vision loss. The main purpose of this study was to evaluate the QoL of people with visual impairments through a questionnaire and identify issues concerning everyday life in the urban and extra-urban areas of Turin.

**Patients and methods:**

A personalized questionnaire including 25 questions was distributed to 100 enrolled patients. It was designed by integrating the most widely used questionnaires related to the QoL of people with visual impairment with questions concerning the city of Turin. The inclusion criteria were any degree of visual impairment (from mild defect to complete blindness), according to Law n. 138/2001 classification. The exclusion criteria were mental disability and residence in care homes. Finally, statistical analysis was performed. Pearson’s Chi-Square test was used to evaluate the strength of the association between two qualitative variables in different sections of the questionnaire. The results were classified as statistically significant with a *p*-value of ≤0.05 or borderline (0.05 < *p*-value<0.10).

**Results:**

Based on responses to question 7 (Q7), 67% of selected patients stated that sight markedly influences their QoL. Moreover, 49% of patients responding to question 12 considered themselves almost completely dependent on other people regarding mobility and movement in and around Turin. In total, 57% used public transport (Q13); however, 50% of them found it challenging to access (Q14). Personal aids (e.g., white cane and magnifying glasses) were adopted only by 51% (Q15), and 63% of patients responding to question 18 suggested a refinement of urban aids (e.g., road signs). Of the 53 patients, 30 patients (56.6%) considered Turin a livable city for visually impaired people (Q19); however, 44 patients (84.6%) reported no significant improvements in Turin’s urban logistics during the last 5 years and highlighted the urgent need to improve urban aids (Q21). Furthermore, the statistical associations studied showed that the loss of vision plays a significant role in influencing the perception of one’s QoL (association of questions 7 and 8, X2 = 112.119, Cramer’s V = 0.548, *p*-value <0.001). In addition, it is more difficult for visually impaired patients living outside the city to move outdoors (Chi-Square = 10.637, Cramer’s V = 0.326, *p* − 245 value = 0.031) and to cross the street (Chi-Square = 14.102, Cramer’s V = 0.376, p-250 value = 0.007). Finally, those who feel independent perceive their lives to be more fulfilling (Chi-Square = 268, X2 = 37.433; Cramer’s V = 0.306, *p* value = 0.002).

**Conclusion:**

Our study showed how vision loss plays a remarkable role in influencing the perception of one’s QoL. Furthermore, it highlighted how the implementation of mobility and the use of personal aids for living in a city, such as Turin, were associated with a better perception of QoL by visually impaired patients. However, it is necessary to improve urban technological development according to the needs of people with visual disability.

## 1 Introduction

The improvement of healthcare worldwide and the growing interest of both individuals and society in the prevention of diseases affecting the visual system are undoubted (1). To enhance and maximize the achievements that have been made over the past 20 years, in 2020, the World Health Organization (WHO) and the International Agency for the Prevention of Blindness gave birth to the VISION 2020 project, The Right of Sight (2, 3). This program aims to eradicate the preventable causes of blindness. The implementation of this project is grounded on data regarding the prevalence of blindness and severe visual impairment caused by treatable diseases, which are still alarmingly high despite the efforts involved. In fact, as early as 2015, the Global Vision Database was created by The Vision Loss Expert Group (VLEG) to gather and analyze information on the epidemiology of low vision around the world. The data collected contributed to the publication of the WHO’s World Report on Vision in 2019. In light of this data, there is an absolute need for the continuation of the plans outlined to drastically reduce the number of cases recorded. According to this study, 2.2 billion people are affected by some degree of visual impairment, and half of them have a preventable and treatable condition (1).

However, what is meant by visual impairment status? The International Classification of Diseases (ICD) classification, drawn up by the WHO, distinguishes vision disorders into two groups: far vision and near vision disorders. The former are sub-classified into four classes according to descending order of severity: blindness, severe, moderate, and mild low vision. Visual acuity of less than 3/60 defines a condition of blindness, while visual acuity values between 3/60 and 6/18 represent severe (range: 3/60–6/60) and moderate (range: 6/60–6/18) low vision, respectively. Finally, mild visual impairment is equivalent to values between 6/18 and 6/12. Therefore, under this classification, values above 6/12 distinguish good visual quality. However, concerning the differentiation of near vision diseases, these are defined by a near visual acuity poorer than N6 (scale and metric notation) or M.08 (scale and metric notation) at 40 cm (4).

Still, in 2020, another analysis, the Global Burden of Disease Study, allowed us to estimate the worldwide prevalence of blindness and visual impairment: 43.3 million people were blind, and 295 million people were affected by moderate-to-severe visual impairment. In addition, it has been predicted that the amount will be set to rise dramatically (5). Dissecting in more detail the etiopathogenesis of these conditions, it has also been demonstrated that the leading causes of moderate-to-severe vision impairment (MSVI) are uncorrected refractive error (86.1 million cases), cataract (78.8 million cases), age-related macular degeneration (AMD) (6.2 million cases), glaucoma (4.1 million cases), and diabetic retinopathy (2.9 million cases) (5, 6).

These statistics are worrying, as they reflect a society plagued by significant visual deficits, not only in terms of health in the strict sense but also in terms of perceived quality of life. The WHO defines health as “a state of physical, mental, and social well-being and not merely the absence of disease” (7), while QoL is referred to as “an individual’s subjective perception of his or her position in life, in the context of a culture and set of values in which he or she lives, in relation to his or her goals, expectations, and concerns” (8). Therefore, a person’s quality of life plays a key role in determining their health status, particularly mental and social health, but undoubtedly, it also reflects on the physical aspect, representing a pivotal point in defining and realizing the individual as a “healthy” person. People with visual impairment complain of severe limitations in performing daily activities that, for a healthy person, are almost taken for granted, such as crossing streets, going outdoors alone, cooking, dressing, and taking care of one’s personal hygiene. As a matter of fact, they encounter greater daily impediments to the movement. Moreover, being blind inevitably makes such individuals more vulnerable to trauma, both physical (due to falls and loss of balance) and social, effectively exposing them more to scams and robberies. In addition, it has been shown that they have an increased risk of developing social withdrawal and depression than healthy population (9), and low vision can cause or exacerbate poverty through reduced employment prospects and work productivity, as well as can negatively affect educational opportunities (10–16). Moreover, not to be overlooked is the isolation that results from the virtual reality in which today’s society is now more and more immersed (social media, dating apps, and forums). Although technologies are state-of-the-art, sight in this sphere is preponderant, and the drastic increase in interpersonal relationships online limits the use of the remaining senses (hearing, smell, and touch), which are instead indispensable for visually impaired people to interact with the outside world. This aspect is even more pronounced and impactful in visually impaired adolescents, given the preponderant virtual involvement of their peers.

However, in addition to affecting the single individual, it all has an indispensable impact on the community to which these individuals belong, given their gradually increasing prevalence, the management has important political, social-health, and economic implications. The resulting distress leads to direct costs (e.g., medical treatments and access to hospital facilities) and indirect expenses (such as loss of workforce and productivity) that inevitably affect society. Such conditions are even more critical in developing countries, among which Africa and Southeast Asia count the most dramatic rates (6, 17, 18). However, the proportion of vision-disabled people is conspicuous even in developed countries. For example, as far as Italy is concerned, according to “Istituto Nazionale di Previdenza Sociale,” National Social Welfare Institute in Italy (INPS) data for 2021, there were 108,856 disabled, blind people and 7,173 residents of Piedmont (19). In Italy, the classification and quantification of visual impairments was established by Law n. 138/2001: “Classification and quantification of visual impairment and standards for ocular examinations” (19, 20), which classifies individuals with visual impairment into four categories as reported in Table 1.

**Table 1:** Italian classification of visual impairments—law no. 138/2001.

	Category	Definition
1.	Totally blind	Total loss of vision in both eyes *or* mere perception of shadow and light or hand motion in both eyes or the better eye *or* binocular residual perimeter less than 3%.
2.	Partially blind	Residual vision not exceeding 1/20 in both eyes or the better eye, even with correction *or* residual binocular perimeter less than 10%.
3.	Severe visual impairment	Residual vision not exceeding 1/10 in both eyes or the better eye, even with correction *or* residual binocular perimeter less than 30%.
4.	Moderate visual impairment	Residual vision not exceeding 2/10 in both eyes or in the better eye, even with a possible correction *or* residual binocular perimeter less than 50%.
5.	Mild visual impairment	Residual vision not exceeding 3/10 in both eyes or the better eye, even with a possible correction *or* residual binocular perimeter of less than 60%.

In light of this background evidence, the main purpose of our observational study is to analyze the quality of life (QoL) of people living with blindness or different grades of visual impairments through a specific and comprehensive questionnaire. It was created *ad hoc* after reviewing the questionnaires made and published previously. Indeed, in the literature, a series of questionnaires have been provided to visually impaired patients to outline their quality of life. The most relevant of those present were Low Vision Quality of Life (LVQoL, designed by Wolftsonn and Cochrane in 2000) (21), National Eye Institute Visual Function Questionnaire (NEI-VFQ) composed of 51 items, and its shortened version of 25 items (NEI-VFQ-25) (22), The Impact of Vision Impairment Questionnaire (IVI) (23), and Visual Function Index 14 (VF − 14) (24). LVQoL was taken as a model as it was constructed to quantify the QoL of visually impaired patients effectively by avoiding unnecessary and redundant questions (21); however, it was focused on collecting data for managing the rehabilitation of such patients, which was not our primary purpose. Our aim was to focus on the repercussions of visual impairment on psychophysical and social facets, i.e., on the QoL of patients. The NEI VFQ-25 is short and simple to administer and provides user-friendly scoring guidance (22). Nevertheless, the ease of execution leads to difficulties in the interpretation of results and quantification of QoL by the clinician, as well as the IVI, consisting of 32 items that are developed to measure the impact of visual impairment on daily routines (23). We considered the original version of the questionnaire from which the NEI VFQ-1 is derived, the NEI VFQ-51, which is extremely time-consuming and inquisitive to be filled out effectively by our patients. VF-14 is based on 14 daily activities (e.g., reading a newspaper, watching TV, or taking part in activity-based activities) that may be affected by cataracts (24), but it does not investigate the influence of other diseases, instead represented in our sample. Moreover, the questionnaires reviewed did not focus on the accessibility of urban centers for visually impaired patients, a central issue for us in affecting the quality of life. In particular, none of the questionnaires had sections concerning Turin, a city taken as a model for investigating critical issues specific to urban centers. Therefore, we created our own *ad hoc* questionnaire, adding specific questions inherent to the city of Turin. The data analysis we collected highlighted accurate, critical points, specifically relating to the mobility of these patients and the utilization of personal aids (e.g., white cane and mobile phone apps) and how they enhance autonomy in movements.

The ultimate aim of our investigation was to gather suggestions on changes and implementations to be introduced in the urban planning of the city of Turin. Although it has a moderate town size compared to other Italian urban cities and foreign metropolises, it could be a potentially good model for the user-friendly renewal of urban centers (25). As mentioned above, the sense of sight is critical for interacting with the outside world and an integral part of society. It is now well-known that urban cities were originally designed without taking into account physical disabilities, particularly visual ones, including crossing the street, stepping on the sidewalk, and avoiding obstacles, which turn out to be extremely challenging and stressful activities for visually impaired people to perform independently. Therefore, enhancing the accessibility of urban centers would contribute to both the physical and especially psychological wellbeing of these individuals.

## 2 Materials and methods

In the first instance, questionnaires reported in the literature about the QoL of people with visual impairments were analyzed: LVQoL (21), NEI-VFQ-25 (22), IVI (23), and VF-14 (24). We constructed a questionnaire to investigate patients’ QoL because it is an independent instrument of the examiner’s judgment and quantifies the patient’s perceived difficulties in performing daily activities. We created our own survey integrating those previously mentioned with elucidations concerning patient mobility in the city of Turin. The items were then evaluated by a multidisciplinary low-vision rehabilitation team consisting of ophthalmologists, trainees in ophthalmology, orthoptists, optometrists, and visually impaired people for relevance. Questions and topics not considered relevant in terms of QoL, autonomy, mobility, and accessibility by the majority of people with visual impairments surveyed were excluded.

For the assessment of internal consistency, both the item-total correlation (correlation of a single item with its subscale without that item, which should be above the value of 0.2) (26) and Cronbach’s α (a measure of internal consistency, which should be between 0.70 and 0.90) (27) were calculated. The item-total correlation was higher than 0.2 for all questions included, and Cronbach’s α was 0.81 for the entire questionnaire. To assess the reliability of the questionnaire, we conducted a test–retest analysis. The Pearson correlation coefficient was higher than 0.80 (0.84), which indicates good reliability of the test.

Our final questionnaire consisted of 25 questions and this is arranged in three sections—sight and quality of life, mobility and autonomy, livability and suggestions for the city of Turin—and an additional one containing general questions, such as age, sex (male/female, M/F), acquired and/or hereditary diseases, the age of onset or diagnosis of them, etiology, visual impairment level (according to Law n. 138/01), and residence (Turin/elsewhere). All procedures in this study concerning conduction and documentation were performed in conformity with the ethical principles set out in the Helsinki Declaration and its revisions and were in accordance with the ethical standards of the committee responsible for human research (institutional and national). No previous ethical approval has been obtained for this survey since it is not required by the institutional committee (A.O.U. Città della Salute e della Scienza di Torino) for non-interventional studies. Consent to participate was obtained in written form and has been registered for all subjects of this study. We decided to use different types of answers according to the degree of precision and the type of information we wanted to obtain to be as functional as possible for our purpose. Most of the questions required closed-end answers on a Likert scale because we considered they provide stratified answers and, at the same time, a better interpretation and analysis of them. However, for some, we preferred the “yes/no” option for question 6 (Q6) and for some mobility and autonomy questions in Section II (Q11, Q13, Q15, Q16, and Q17). This was either to simplify the interpretation of the results (e.g., in Q6, it was more important for us to know whether the visual impairment affects interpersonal relationships and not so much the extent of this impact, or as for question 13, whether they use public transport) or because the answer on a Likert scale was not applicable (e.g., Q11 concerning the need or otherwise of accompaniment in carrying out daily activities). Finally, questions 9, 10, 24, and 25 required open-ended answers. In the first two, the aim was to give the patient more freedom of expression and ensure greater accuracy in the answer. On the other hand, questions 24 and 25 were designed on an *ad hoc* basis to stimulate patients to give advice, express criticism, and elaborate suggestions on improving the city of Turin in terms of accessibility, a central topic of our survey and not present in the previously mentioned questionnaires.

Second, we selected patients from the Ophthalmology department at the University of Turin: a previous comprehensive eye examination with accurate visual acuity measurement and a thorough medical history was an imperative criterion for including patients in the study. These data were collected digitally in the medical records in the clinic archive (TrakCare Informatic System). The exclusion criteria were incomplete visits recorded in the operating system, a history of mental disability, and the lack of quantitative assessment of visual acuity in the medical record mentioned above. Patients residing exclusively in hospital facilities (e.g., retirement homes) and/or unable to understand or answer the questionnaire were also excluded from the study. To construct the study sample, the simple random sampling method was chosen to select 100 patients from those in the database with characteristics corresponding to the criteria described above (978 patients). Informed consent was obtained from subjects participating in the experiment. The questionnaire was read to each patient and completed with the responses of the latter in the presence of a witness. To ignite adherence to completion, a collaboration was started with the Italian Union of the Blind and Visually Impaired people of Turin.

Finally, all the data collected were processed into a matrix. The program we used was Microsoft Office Excel 365 version 16.60. The statistical analysis was conducted using the R program, developed by the R Development Core Team version 4.2.0 and the IBM SPSS Statistics for Windows, Version 28.0 program; IBM Corp., Armonk, New York, USA.

**Figure 1:**
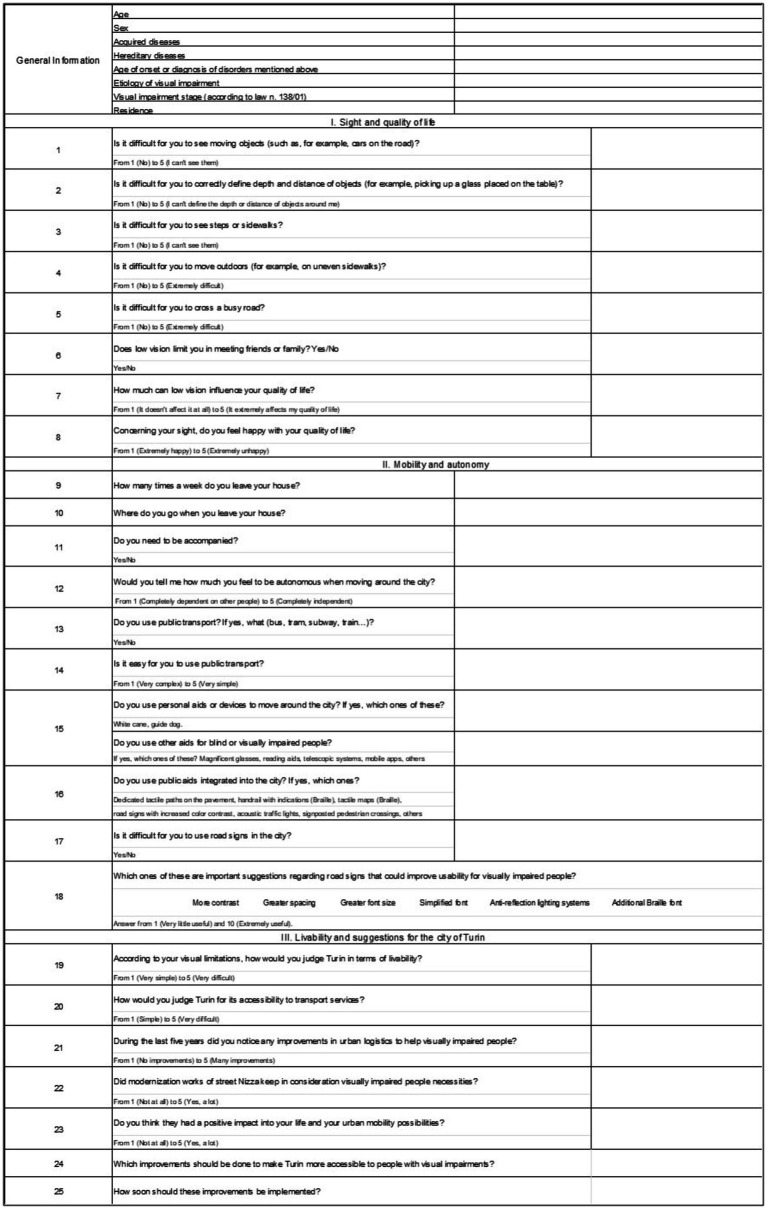
Layout of the questionnaire submitted to study patients.

The descriptive statistic was reported as a range of continuous variables, along with mean and standard deviation (SD), while as frequency or percentage for categorical variables of different sections of the questionnaire. Pearson’s Chi-Square test was used to evaluate the strength of association between two qualitative variables. The results were classified as statistically significant with a *p*-value of ≤0.05, borderline (0.05 < *p*-value<0.10), or not statistically significant with a *p*-value of >0.05. When necessary (>20% of values ≤5 and/or presence of values <1) and in order to have more easily interpretable data, Cramer’s V test was used. We reported the questionnaire submitted to patients in its entirety in Figure 1.

## 3 Results

### 3.1 General information

**Figure 2:**
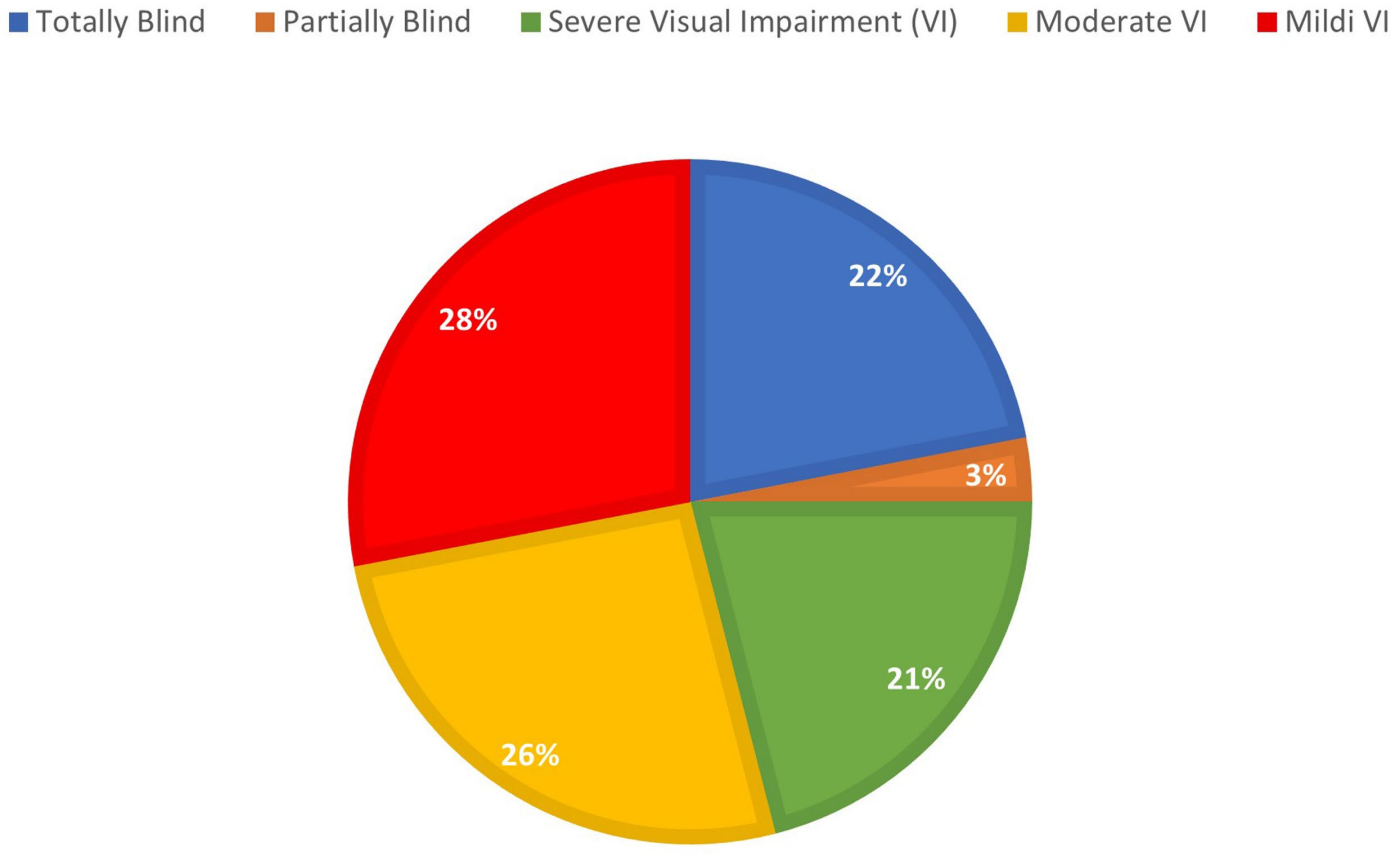
Percentage distribution of visual impairment in the study sample.

This section aimed to collect baseline demographics and ocular features of the patients included in the study. It is shown in Table 2 and Figures 1, 2.

**Table 2:** Baseline demographics and ocular features of the patients included.

Demographic/characteristic	Data
Age (years) (mean ± SD) (range)	74.9 ± 13.6 (25–75)
Sex (%)	
Male	46%
Female	54%
Residence (%)	
Turin	49%
Elsewhere	51%
Ocular disease (%)	
Congenital	10%
Acquired	90%
Acquired disease onset or diagnosis (years) (mean ± SD)	68.8 ± 14.37
Visual impairment level	
Totally blind	22%
Partially blind	3%
Severely visually impaired	21%
Moderately visually impaired	26%
Slightly visually impaired	28%
Low vision etiology (*n*)	
Age-related macular degeneration	46
Diabetic retinopathy	16
Glaucoma	6
CNV	6
Corneal diseases	5
Retinitis pigmentosa	4
Other macular disorders	4
Ocular trauma	3
Optic nerve and neurological diseases	2
High myopia	2
Retinal vascular disorders	2
Sarcoidosis	1
Cataract	1
Ocular neoplasia	1

### 3.2 First section

The first section of the questionnaire aims to understand the main difficulties associated with visual impairment in daily activities and the quality of life of enrolled patients. This section consists of 8 questions with numerical responses from 1 to 5, except for question 6 (“Does low vision limit you in meeting friends or family?”) with a binary response (yes/no). To this last question, 66% of responders replied “no,” while 34% answered “yes.” In this part of the questionnaire, 27 and 40% of the sample stated that vision affects their QoL (Q7) markedly (score 4) and extremely significantly (score 5), respectively, but only 15 and 5% asserted to feel unhappy (score 4) or extremely unhappy (score 5) about their QoL (Q8), respectively (Table 3).

**Table 3:** The results of the questionnaire first section.

Question	1	2	3	4	5	Total
1. Is it difficult for you to see moving objects?	14	38	25	15	8	100
2. Is it difficult for you to correctly define depth and distance of objects?	4	37	26	23	10	100
3. Is it difficult for you to see steps or sidewalks?	8	29	31	19	13	100
4. Is it difficult for you to move outdoors?	12	21	33	23	11	100
5. Is it difficult for you to cross a busy road?	7	31	31	14	17	100
7. How much can low vision influence your QoL?	1	7	25	27	40	100
8. Concerning your sight, do you feel happy with your QoL?	1	40	39	15	5	100

### 3.3 Second section

The second section of the questionnaire focuses on patients’ mobility and autonomy. The purpose of this section was to understand the degree of self-sufficiency in moving and performing normal daily activities of patients with visual impairment. This section includes 10 questions, from 9 to 18 (Q9–Q18). Q9 and Q10 required open answers, while the responses to Q12 and Q14 were on a Likert scale. The remaining questions required “yes/no” answers (Q11, Q13, Q15, Q16, and Q17). More than half of the interviewees (54%) referred to leaving their houses from 4 to 7 times a week (Q9); the other 46% reported going out less than 4 times a week, and 5% of them almost never leave their homes except for commitments that cannot be postponed (Q9). Moreover, 44% of all the patients need at least one companion to be able to move outside (Q11), and 49% of all interviewees answered 1 or 2 to Q12, considering themselves completely or almost completely dependent in movement on other people. Another goal of this section was to investigate public transportation use by these patients. The majority of interviewees (57%) use public transport (Q13); unfortunately, 50% of them find it challenging to access it (Q14). Regarding the utilization of personal aids, only 51% of the subjects examined declared to adopt them: 19 people use a white cane, 18 help themselves in near-distance activities with lens magnifying glasses, 13 use mobile phone apps for mobility and reading with vocal synthesizers, and only 1 person has a personal guide dog (Q15). Instead, in Q16, we investigated which aids integrated in urban planning are more often used by interviewees. The most used are acoustic traffic lights (20%), followed by tactile sidewalk paths (13%) and maps written in Braille (3%). Finally, in Q17, it was asked whether it was difficult for the interviewees to see road signs, where 76% of the interviewees responded with difficulties in seeing them. In Q18, we instead asked which improvements could be performed for road signs, for which, 63% of them responded that an increase in the size of the signs could be useful, also greater lighting (27%), anti-reflection systems (25%), and an increment in color contrast (16%). In addition, more than two-thirds of the patients (68.9%) considered these proposals “useful” to “extremely useful.”

### 3.4 Third section

**Figure 3:**
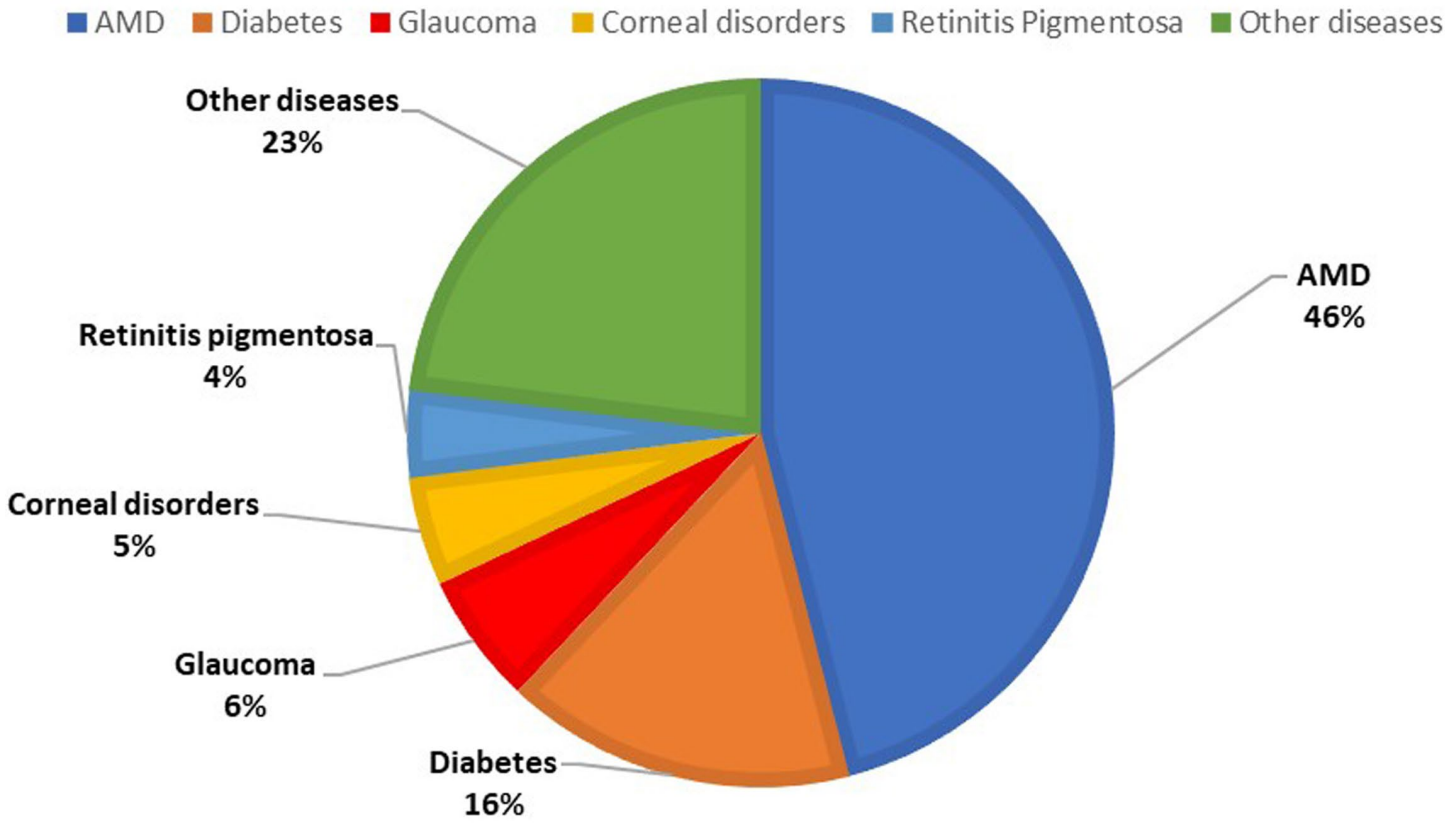
Percentage distribution of ocular diseases in the study sample.

The third section of the questionnaire concerns livability and mobility in Turin. This section includes seven questions, from 19 to 25 (Q19–Q25). The responses to the first five questions (Q19–Q23) were to be recorded on a Likert scale, while the last two questions (Q24–Q25) required open answers. Indeed, these include suggestions to improve Turin’s accessibility to visually impaired people. No one answered “very difficult to live in” in Q19, where 30 patients out of 53 (56.6%) considered Turin a livable or very livable city for visually impaired people; however, only 28.8% of the interviewees gave “4” or “5” as answers to Q20. Low satisfaction rates were expressed about improvements in Turin’s urban logistics for blind and visually impaired people during the last 5 years (Q21), where 44 of 52 patients (84.6%) reported low or no significant improvements. The results are shown in Table 4. Regarding questions Q24 and Q25, we created five macro-categories as possible answers: road surface improvements, electric cars and scooters, audible traffic lights, public transport, and street lighting at night and are reported in Table 5. Of the 100 subjects in the sample, 68 responded to question 24. Each interviewee could give multiple suggestions at the same time. In total, 53 respondents, 77.8% of the total, stated that the road surface needs improvement. On the other hand, 39 out of 68 respondents, 57.4% of the total, warned of the hazards posed by electric vehicles. The other tips were increasing acoustic traffic lights (27.9%), public transportation services (17.6%), and nighttime street lighting (3.3%). Finally, to question 25, the vast majority of the 68 interviewees assumed that these improvements should be implemented as soon as possible (84%) (see Figures 2, 3).

**Table 4:** The results of the questionnaire third section.

Question	1	2	3	4	5	Total
19. According to your visual limitations, how would you judge Turin in terms of livability?	0	6	17	28	2	53
20. How would you judge Turin for its accessibility to transport services?	2	13	17	11	2	45
21. During the last 5 years, did you notice any improvements in urban logistics to help visually impaired people?	35	9	7	0	1	52
22. Did renovation works of Turin streets keep in consideration visually impaired people’s necessities?	10	4	3	1	1	19
23. Do you think they had a positive impact on your life and your urban mobility possibilities?	5	4	2	1	1	13

**Table 5:** Five macro-categories and suggestions from inpatients enrolled (% answers).

Suggestions for improving accessibility of Turin			
	Categories	Suggestions	68 responders
1	Road surface improvements	Sidewalks renovation, architectural barriers removal, and steps replacement with ramps	77.8%
2	Electric cars and scooters	Need to establish parking and use regulations	57.4%
3	Audible traffic lights	Increase accessibility, remote activation, and the number of traffic lights	27.9%
4	Public transport	Service improvement	17.6%
5	Increase of street lighting at night		3.3%

### 3.5 Statistical analysis

Once our questionnaire was distributed for this study including 100 patients, statistical associations were made between the various sections of the questionnaire to analyze both mobility and QoL of visually impaired people. Since these were qualitative variables, the Pearson Chi-Square test was carried out, and data were shown schematically in the following tables (6–12). We analyzed the first association between living in Turin and the answers to question 4 “Is it difficult for you to move outdoors?” It turned out to be statistically significant (Chi-Square = 10.637, Cramer’s V = 0.326, *p*-value = 0.031). This association, applying the adjusted residual, was significant, especially for people who voted 1 (“not difficult”) and lived in Turin (83.3% versus 16.7%). On the other hand, most of the patients who voted 5 (“extremely difficult”) reside outside of Turin (adjusted residual = +1.5) (Table 6).

**Table 6:** Association between question 4 and residence (or not) in Turin.

Question 4				
Answer		Resident in Turin		Total
		No	Yes	
1	Responders	2	10	12
	%	16.7%	83.3%	100%
	Adjusted residual	-2.5	2.5	
2	Responders	8	13	21
	%	38.1%	61.9%	100%
	Adjusted residual	−1.3	1.3	
3	Responders	20	13	33
	%	60.6%	39.4%	100%
	Adjusted residual	1.3	−1.3	
4	Responders	13	10	23
	%	56.5%	43.5%	100%
	Adjusted residual	0.6	−0.6	
5	Responders	8	3	11
	%	72.70%	27.30%	100%
	Adjusted residual	1.5	−1.5	
TOTAL	Responders	51	49	100%
	%	51%	49%	100%

The chi-square statistic is 10.6369. The *p*-value is 0.030962. The result is significant at *p* < 0.05.

Then, we found a statistically significant association (Chi-Square = 14.102, Cramer’s V = 0.376, p-value = 0.007) between residence in Turin and the answers to question 5: “Is it difficult for you to cross a busy road? From 1 (No) to 5 (Extremely difficult).” This association, applying the adjusted residual, gets more significant, especially for people who voted 5 (“extremely difficult”) and do not live in Turin (82.4% versus 17.6%) (Table 7). Therefore, these first two associations showed how interviewees who do not live in Turin perceive walking outdoors and crossing a busy road much more difficult than Turin citizens. In addition, the association between question 7 “How much can low vision influence your quality of life? From 1 (It does not affect me at all) to 5 (It extremely affects my quality of life)” and question 8 “Concerning your sight, do you feel happy with your QoL? From 1 (extremely happy) to 5 (extremely unhappy)” was analyzed. This analysis resulted in a statistically significant association (Chi-Square = 112.119; Cramer’s V = 0.548; *p*-value<0.001) (Table 8). In particular, applying the adjusted residual, the statistical association is stronger for people who voted 5 on both questions; the same principle is applicable to those who answered 2 on both questions. What does this mean? It means that patients who consider that low vision greatly affects their routine perceive their QoL to be low, while interviewees who think that sight has little influence on their QoL are satisfied with it. Another attractive correlation to study was between question 8 “Concerning your sight, do you feel happy with your QoL?” and question 12 “Define how autonomous you consider yourself to be in moving around the city from 1 (completely dependent on other people) to 5 (completely independent).” It was found to be statistically significant (Chi-Square X2 = 37.433; Cramer’s V = 0.306, *p* value = 0.002), especially for those who gave a low score to question 8 (who therefore have a good/very good perception of their own QoL) and those who gave very low (vote = 1 for 7.5%) or very high (vote = 5 for 25%) scores to question 12 (Table 9). Therefore, it shows that people who have a good perception of their lives feel strongly independent at the same time. Another investigated association was between question 8 “Concerning your sight, do you feel happy with your QoL?” and being a Turin citizen, it was instead found to be statistically borderline (Chi-Square = 8.394; Cramer’s V = 0.290, *p*-value = 0.078) as the *p*-value was between 0.05 and 0.10 (Table 10). This one, based on the adjusted residual, is stronger for people who voted 5 (“extremely unhappy”) and are not living in Turin, suggesting that people who do not live in Turin have a worse perception of their QoL. Moreover, we analyzed another correlation between residing in Turin and question 14 “Is it easy for you to use public transport? From 1 (very complex) to 5 (very simple).” The association was not statistically significant (*p*-value = 0.761). This suggests that there is probably no perceived difference between citizens in Turin and those in suburban areas in terms of accessibility to use public transportation. On the other hand, concerning personal aids, the most used by the interviewees was white cane (19%). A statistically significant association was found between people’s perception of their QoL and the use of a white cane (Chi-Square = 9.460; Cramer’s V = 0.499, *p*-value = 0.05). The use of a white cane was also related to question 14, and this association was statistically significant (Chi-Square = 9.829; Cramer’s V = 0.509; *p*-value = 0.043), suggesting that its use helps to perceive public transport fruition as easier. Finally, regarding suggestions given by the interviewees in questions 24 “Which improvements should be done to make Turin more accessible to people with visual impairments?” and 25 “How soon should these improvements be implemented?,” the only statistically significant association was found between residence in Turin and have placed the public transport improvement among the main requests (Chi-Square = 5.922; Cramer’s V = 0.295; *p*-value = 0.015) (Table 11). This could suggest that people living in Turin perceive this as an urgent need.

**Table 7:** Association between residence in Turin and answers to question 5.

Question 5				
Answer		Resident in Turin		Total
		No	Yes	
1	Responders	2	5	7
	%	28.6%	71.4%	100%
	Adjusted residual	-1.2	1.2	
2	Responders	12	19	31
	%	38.7%	61.3%	100%
	Adjusted residual	-1.6	1.6	
3	Responders	19	12	31
	%	61.3%	38.7%	100%
	Adjusted residual	1.4	-1.4	
4	Responders	4	10	14
	%	28.6%	71.4%	100%
	Adjusted residual	-1.8	1.8	
5	Responders	14	3	17
	%	82.4%	17.6%	100,0%
	Adjusted residual	2.8	−2.8	
TOTAL	Responders	51	49	100%
	%	51%	49%	100%

The chi-square statistic is 14.1017. The *p*-value is 0.006977. The result is significant at *p* < 0.05.

**Table 8:** Association between answers to questions 7 and 8.

	Question 8						
	Answer		1	2	3	4	5

Question 7	1	Responders	1	0	0	0	0
		% Q7	100%	0%	0%	0%	0%
		Adjusted residual	10	−0.8	−0.8	−0.4	−0.4
	2	Responders	0	6	1	0	0
		% Q7	0%	85.7%	14.3%	0%	0%
		Adjusted residual	−0.3	2.6	−1.4	−1.2	−0.6
	3	Responders	0	14	9	2	0
		% Q7	0%	56%	36%	8%	0%
		Adjusted residual	−0.6	1.9	−0.4	−1.1	−1.3
	4	Responders	0	9	11	7	0
		% Q7	0%	33.3%	40.7%	25.9%	0%
		Adjusted residual	−0.6	−0.8	0.2	1.9	−1.4
	5	Responders	0	11	18	6	5
		% Q7	0%	27.5%	45%	15%	12.5%
		Adjusted residual	−0.8	−2.1	1	0	2.8
	TOTAL	Responders	1	40	39	15	5
		% Q7	1%	40%	39%	15%	5%

The chi-square statistic is 112.119. The *p*-value is 0.001. The result is significant at *p* < 0.05.

**Table 9:** Association between answers to questions 12 and 8.

	Question 12						
	Answer		1	2	3	4	5

Question 8	1	Responders	0	0	0	1	0
		% Q7	0%	0%	0%	100%	0%
		Adjusted residual	−0.6	−0.6	−0.5	2.1	−0.4
	2	Responders	3	12	5	10	10
		% Q7	7.5%	30.0%	12.5%	25%	25%
		Adjusted residual	−3.2	−0.9	−1.4	1.2	2.9
	3	Responders	12	12	10	4	1
		% Q7	30.8%	30.8%	25.6%	10.3%	2.6%
		Adjusted residual	1.3	1.1	1.4	−1.8	−2.5
	4	Responders	6	1	4	4	0
		% Q7	40%	6.7%	26.7%	26.7%	0%
		Adjusted residual	1.6	−1.8	0.8	0.8	−1.6
	5	Responders	3	0	0	0	2
		% Q7	60%	0%	0%	0%	40%
		Adjusted residual	1.9	−1.3	−1.1	−1.1	1.8
	TOTAL	Responders	24	25	19	19	13
		% Q7	24%	25%	19%	19%	13%

The chi-square statistic is 37.433. The *p*-value is 0.002. The result is significant at *p* < 0.05.

**Table 10:** Association between residence in Turin and answers to question 8.

Question 8				
Answer		Resident in Turin		Total
		No	Yes	
1	Responders	0	1	1
	%	0.0%	100.0%	100%
	Adjusted residual	−1	1	
2	Responders	16	24	40
	%	40.0%	60.0%	100%
	Adjusted residual	−1.8	1.8	
3	Responders	21	18	39
	%	53.8%	46.2%	100%
	Adjusted residual	0.5	−0.5	
4	Responders	9	6	15
	%	60.0%	40.0%	100%
	Adjusted residual	0.8	−0.8	
5	Responders	5	0	5
	%	100.00%	0.00%	100%
	Adjusted residual	2.2	−2.2	
TOTAL	Responders	51	49	100%
	%	51%	49%	100%

The chi-square statistic is 8.394. The *p*-value is 0.078. The result is not significant at *p* < 0.05, however, it is significant at *p* < 0.1.

**Table 11:** Association between public transport users and residents in Turin.

Public transport		Resident in Turin		Total
Users		No	Yes	
No	Responders	31	25	56
	%	55.4%	44.6%	100%
	Adjusted residual	2.4	−2.4	
Yes	Responders	2	10	12
	%	16.7%	83.3%	100%
	Adjusted residual	−2.4	2.4	
Total	Responders	33	35	68
	%	48.5%	51.5%	100%

The chi-square statistic is 5.922. The *p*-value is 0.015. The result is significant at *p* < 0.05.

## 4 Discussion

In recent decades, the attention of social and health institutions has focused heavily on developing strategies and programs to fight disorders that cause visual impairment. In fact, more than two million people have vision problems worldwide, and often the causes are preventable and treatable (1). On the other hand, medicine goals have expanded from simply studying, diagnosing, and treating diseases from a purely clinical-biological perspective: the new frontier is to study the role that diseases have on the psychological and emotional traits of patients. Indeed, the patient’s biological disorder is not only clinical but has socio-emotional implications on their quality of life (28, 29). We share the interest and perception that therapeutic horizons should be broadened by considering the patient in their psychophysical whole and not only by analyzing the visual deficit from a clinical point of view. In fact, we consider this aspect to be of fundamental importance and that, despite the progress made in this regard, it is still poorly investigated and consequently underestimated. Each individual is a union of mind and body, and matter and soul, and the emotional and psychological implications of a disease on one’s individuality and health play a still little-known role in the course and therapeutic management of the disease itself. This becomes easier to understand when one considers how total or partial vision loss affects all activities performed in daily life and inevitably affects the quality of the latter (30); in fact, the onset of depressive and anxiety disorders is frequent in these patients (31). Moreover, the social and economic costs cannot be overlooked, especially since this disability is so widespread and prevalent (27). For this reason, it is desirable for society to become properly aware of and interested in investigating and managing the challenges faced by these individuals. In light of this, the purpose of this study was to help investigate the role of visual impairment-causing visual diseases on patients’ quality of life and their perceived autonomy in movement and daily activities. In this regard, we considered it relevant to investigate the accessibility to urban centers by these patients, particularly the city of Turin. In our opinion, such a medium-sized city can be considered a good model for studying the quality of life of visually impaired people and serve as an example for improving accessibility to services by reorganizing and restructuring urban centers, even large ones. As a survey instrument, we created our own validated questionnaire inspired by those published in the literature on this topic and focused on the accessibility of Turin. We recruited and distributed the questionnaires to 100 patients. Regarding the section on general information (Table 2), visual impairment was caused in most of these patients by age-associated maculopathy (46%), confirming data recorded in Europe (32). On the other hand, among the diseases that cause blindness, cataracts (94.0 million cases) and uncorrected refractive error (86.1 million cases) are present worldwide (6), and there are no cases in our study sample. Thus, they were underrepresented in our study group. This discrepancy is related to the accessibility of early examinations and treatment in developed countries, such as Italy, whereas this is not guaranteed in developing countries. In fact, they are plagued by these preventable diseases, yet not managed in time due to a lack of resources.

Regarding the first section of the survey, it was inherent to the perception of one’s own QoL: according to the answers given, visual impairment was found to be decisive in this regard for most of the patients. In fact, it reveals how impaired vision negatively affects the quality of life of patients in the study. Patients who feel that low vision greatly affects their routine perceive their QoL worse, while respondents for whom vision impacts their QoL poorly are more satisfied.

Then, through the questions in the second section, we wanted to investigate the independence in movement and autonomy in conducting daily activities of these patients. More than half of the patients interviewed go out more than four times a week, but a similar percentage need a companion to do this, carry out daily activities (shopping, work, etc.), and feel completely dependent on other people to complete them. This negatively affects their self-perception and their QoL, generating a sense of inadequacy and inability to lead a normal life. This reality is also made more difficult by the demanding use of public transport; in fact, according to the answers given, 57% of the patients interviewed use public transport to move outside their homes, but half of the subjects find this inconvenient. These data suggest an important dissatisfaction with public mobility infrastructures. Moreover, they are an important index both to assess the QoL of these patients and to quantify the degree of accessibility to the city’s facilities. Related to this is the discouraging finding on the use of personal aids, only one-fifth of the people surveyed have a white cane, which is the most commonly used device. The provision of personal devices such as this or mobile phone apps and, even more so, guide dogs, is a need that would help these people to make use of public infrastructures and, above all, to feel more self-sufficient and independent in moving around in everyday life. The sense of independence and autonomy increases self-esteem and greatly improves the quality of life. In addition, urban devices would also increase the freedom of movement of visually impaired patients. The interviewees emphasized that it would be desirable to install more audible traffic lights, road signs with anti-reflection and higher-contrast systems, and maps with tactile indications. Finally, the third section of the questionnaire contains issues concerning the livability and suitability of the city of Turin for visually impaired patients. This section was designed to construct questions that were targeted and comprehensive but, at the same time, easy to respond to. The results showed that most people consider Turin not adequately modernized and modified to make it more accessible for visually impaired people. Therefore, road and infrastructure renovations did not satisfy this category of inhabitants. The final questions (Q24 and Q25) were intended to give the participants more leeway by indicating which improvements they felt were most urgent to implement. Of the various options proposed, improving the road surface emerged as the most important goal to be achieved. Another major problem highlighted is the exponential spread of electric vehicles (cars, motorbikes, scooters, etc.); they produce no sound, so their proximity cannot be perceived by visually impaired people, who mainly use hearing and touch to orient themselves and move around urban spaces. In addition, electric scooters are often parked on sidewalks, hindering pedestrians, especially visually impaired people, and creating severe discomfort. The other proposed topics for improving accessibility were an increase in hourly public transport passages and street lighting. Hence, an overwhelming majority of respondents strongly thought these enhancements should be implemented as soon as possible, further supporting a general perception of urgency for these changes. Unfortunately, only half of the study participants answered the questions in this section. The completion of the questionnaire in its various parts is voluntary, so we do not know the reasons for such low adherence. We assume that a large proportion of the patients, not residents of the city of Turin but living in the neighboring areas, did not respond as they did not consider their answers applicable. In this regard, we analyzed residences in suburban areas of the city. The statistical surveys have shown that the perception of quality of life is poor in patients living in these areas in terms of reduced autonomy in crossing streets and walking outdoors. This may reasonably be due to a lower presence of urban auxiliary devices (audible traffic lights and adequate street lighting) and to a greater degradation and failure to repair the road surface, which is considerably more prone to rare signaling and consequent resurfacing. However, this different perception did not prove to be significant with regard to the usability of public transport, regardless of the location of their homes, the visually impaired patients interviewed experienced similar difficulties. Certainly, in light of this finding, any new surveys will be aimed at improving compliance and further facilitating patient expression in this regard. Despite the constraints and difficulties encountered, the questionnaire submitted provided an overview of the perceptions and adversities faced by visually impaired patients in their everyday lives. Our study, in fact, brought to light serious issues of accessibility in the city of Turin, which, however, can be roughly extended to urban centers in general and the surrounding suburban areas. More accessible cities for visually impaired patients would contribute to greater integration of these individuals into city social activities and, consequently, greater personal satisfaction and fulfillment. A better perception of QoL also seems to influence the course and management of the disease. A patient who feels understood and part of a system that cares about him and his happiness is a more active citizen, spurred on to make the best of his abilities (33). The implications of a low QoL translate into physical-clinical and, above all, psychological discomfort. The depression and consequent isolation of these subjects are documented and described (34, 35), as is the reduced development of integration and support programs for these patients and their families, who are often already economically struggling due to obvious obstacles in employment. Therefore, the concept of accessibility not only concerns the reality of urban and suburban infrastructures but also concerns the achievement of mental, social, and psychological health (29). Therefore, upon diagnosis of these disabling pathologies, a pathway of psychological support and practical support should be set up to request and obtain personal aids, for example, which are often difficult to obtain.

### 4.1 Limitations of the study

Although the sample is heterogeneous in terms of age, sex, and visual impairment level, there are significant differences between the causes of low vision in the study sample compared to global ones. This may be related to the fact that people accessing Eye Clinic University are more often affected by various chronic conditions, such as AMD, glaucoma, and diabetic retinopathy, than those who have uncorrected refractive errors and cataracts (the two leading causes of blindness and low vision worldwide), which represent a consistency part of the prevalence in poor or developing countries, where access to healthcare is lower even for these easily treatable conditions.

The sample size is relevant; however, it is probably small according to the complexity of the problems we analyzed. Therefore, it will be necessary to increase the sample size and make it as heterogeneous as possible to be able to implement fundamental changes for people affected by blindness or visual impairment.

## 5 Conclusion

Overall, our study showed how blindness and low vision are considered relevant in influencing the perception of one’s QoL. Certainly, functional and clinical impairment need to be managed as best as possible in medical and hospital settings, but emotional, social, and educational facets cannot be ignored. Indeed, visual impairment often afflicts individuals by undermining their concept of independence and autonomy. Unfortunately, these aspects have been largely underestimated in the past decades and are still not adequately considered nowadays. In addition, this study revealed that the implementation of mobility, the use of personal aids for movement, and living in cities, such as Turin, are associated with a better perception of QoL by blind or visually impaired patients. However, technological development should aspire to help these individuals to a greater extent. For example, the massive rise in the number of electric vehicles, instead of those with endothermic engines, certainly benefits noise reduction in urban realities. Conversely, their quietness can be very dangerous for those who, instead of sight, are forced to rely on their other senses, including hearing, to get around. The evidence from this study is preliminary; however, it is essential for restructuring existing cities and designing future ones to respect the needs of people with disabilities, particularly those with visual impairment. Future studies could implement our data and help to increasingly improve the quality of life of these patients, which is a critical aspect not only for the individuals but for the wellbeing of society as a whole.

## Data availability statement

The original contributions presented in the study are included in the article/supplementary material, further inquiries can be directed to the corresponding author.

## Ethics statement

Ethical approval was not required for the studies involving humans because it is not required by the institutional committee (A.O.U. Città della Salute e della Scienza di Torino) for non-interventional studies. Consent to participate was obtained in written form and has been registered for all subjects of this study. The studies were conducted in accordance with the local legislation and institutional requirements. The participants provided their written informed consent to participate in this study.

## Author contributions

AN: Conceptualization, Resources, Supervision, Visualization, Writing – original draft, Writing – review & editing. AlB: Data curation, Investigation, Methodology, Writing – original draft. AnB: Data curation, Formal analysis, Writing – original draft. AC: Conceptualization, Writing – review & editing. RN: Project administration, Resources, Supervision, Visualization, Writing – original draft, Writing – review & editing.
